# Brain-targeted intranasal delivery of dopamine with borneol and lactoferrin co-modified nanoparticles for treating Parkinson’s disease

**DOI:** 10.1080/10717544.2019.1636420

**Published:** 2019-07-10

**Authors:** Shengnan Tang, Aiping Wang, Xiuju Yan, Liuxiang Chu, Xiucheng Yang, Yina Song, Kaoxiang Sun, Xin Yu, Rongxia Liu, Zimei Wu, Peng Xue

**Affiliations:** a School of Pharmacy, Collaborative Innovation Center of Advanced Drug Delivery System and Biotech Drugs in Universities of Shandong, Key Laboratory of Molecular Pharmacology and Drug Evaluation (Yantai University), Ministry of Education, Yantai University, Yantai, China;; b State Key Laboratory of Long-Acting and Targeting Drug Delivery System, Shandong Luye Pharmaceutical Co., Ltd, Yantai, China

**Keywords:** Dopamine, lactoferrin, borneol, nose-to-brain targeted nanoparticles, Parkinson’s disease

## Abstract

Efficient delivery of brain-targeted drugs is highly important for successful therapy in Parkinson’s disease (PD). This study was designed to formulate borneol and lactoferrin co-modified nanoparticles (Lf-BNPs) encapsulated dopamine as a novel drug delivery system to achieve maximum therapeutic efficacy and reduce side effects for PD. Dopamine Lf-BNPs were prepared using the double emulsion solvent evaporation method and evaluated for physicochemical and pharmaceutical properties. *In vitro* cytotoxicity studies indicated that treatment with dopamine Lf-BNPs has relatively low cytotoxicity in SH-SY5Y and 16HBE cells. Qualitative and quantitative cellular uptake experiments indicated that Lf modification of NPs increased cellular uptake of SH-SY5Y cells and 16HBE cells, and borneol modification can promote the cellular uptake of 16HBE. *In vivo* pharmacokinetic studies indicated that AUC_0–12 h_ in the rat brain for dopamine Lf-BNPs was significantly higher (*p* < .05) than that of dopamine nanoparticles. Intranasal administration of dopamine Lf-BNPs effectively alleviated the 6-hydroxydopamine-induced striatum lesion in rats as indicated by the contralateral rotation behavior test and results for striatal monoamine neurotransmitter content detection. Taken together, intranasal administration of dopamine Lf-BNPs may be an effective drug delivery system for Parkinson’s disease.

## Introduction

Parkinson’s disease (PD) is the second most common progressive neurodegenerative disorder characterized by deterioration of midbrain nigrostriatal dopaminergic neurons (Catalan et al., 2018; Owens-Walton et al., 2018). The main symptoms of PD include hypokinesia, bradykinesia, rigidity, and resting tremor that correlate with decreased striatal dopamine (Modi et al., 2009; Rodriguez-Nogales et al., 2016). Currently, the frontline drugs for PD include monoamine oxidase B inhibitors, dopamine receptor agonists, and the dopamine precursor levodopa. However, as the disease progresses, patients become less responsive to levodopa due to the loss of 3,4-dihydroxyphenylalanine decarboxylase, an enzyme that converts levodopa to dopamine (Salat & Tolosa, 2013; Krishna et al., 2014). Additionally, the poor blood–brain barrier permeation and drug-release kinetics resulting in peripheral adverse effects limit the effectiveness of current treatment.

A novel approach is to delivery dopamine itself to brain in the form of biodegradable polymeric nanoparticles (NPs) encapsulated dopamine via nose-to-brain delivery to enhance therapeutic efficacy and reduce adverse effects. NPs can increase the stability and bioavailability of dopamine, allowing sustained release of dopamine, and avoid its peripheral metabolism. NPs, owing to their small size, are compatible with various administration routes including intranasal administration. An intranasal nose-to-brain delivery strategy may offer several advantages. Intranasal delivery systems are more efficient in noninvasively delivering drugs to the brain via the olfactory and trigeminal nerves from the nasal olfactory epithelium by circumventing the blood–brain barrier (Haque et al., 2014; Akilo et al., 2016; Mittal et al., 2016; Chu et al., 2018; Md et al., 2013). Direct and brain-targeted drug delivery may allow enhanced therapeutic effects even at a lower drug dose, allow rapid drug absorption at the target site, avoid first-pass elimination, and reduce toxicity (Lalani et al., 2014; Gomes et al., 2018). Several studies on piperine (Elnaggar et al., 2015), tarenflurbil (Muntimadugu et al., 2016), risperidone (Narayan et al., 2016), glycyrrhizic acid (Ahmad et al., 2019) and alginate (Haque et al., 2014) have substantiated the theory of nose-to-brain delivery systems. UPLC/ESI-Q-ToF-MS/MS based bioanalytical method was developed and validated for pharmacokinetics, biodistribution, brain-targeting efficiency and brain drug-targeting potential studies post intranasal (i.n.) administration which showed enhanced bioavailability of drug in brain as compared to intravenous administration (Ahmad et al., 2016; Ahmad et al., 2018; Ahmad et al., 2017a).

Although the intranasal delivery method has many advantages, rapid clearing of drugs or formulation by the mucociliary system in the nose limit its applications. Hence, it is important to develop a newer method that can allow the drug to remain in the nasal cavity for a longer period, for instance, by using chitosan with mucoadhesive nature enhancing the nasal retention time (Ahmad et al., 2017b) or enhancing the permeability across the epithelial membrane (Tzeyung et al., 2019). Borneol is a bicyclic monoterpene and is widely applied to traditional Chinese medicine (Bhatia et al., 2008). It has anti-epileptogenic (Ehrnhofer-Ressler et al., 2013) and anti-inflammatory effects (Tambe et al., 2016). In particular, it can enhance the brain penetration and transport of other drugs to improve bioavailability and enhance the distribution of drugs across various physical barriers such as the nasal mucosa and the blood-brain barrier (Qi et al., 2013). Although borneol is effective in promoting drug transport to the brain (Ren et al., 2013), drugs could still be metabolized at non-targeting tissues, which will cause serious adverse effects. In our previous studies (Bi et al., 2016; Yan et al., 2018), lactoferrin (Lf) was found to be an effective biological ligand for drug delivery to the striatum. The Lf receptor is overexpressed on the apical surface of respiratory epithelial cells and in the capillaries and neurons related to neurodegenerative diseases such as PD and Alzheimer’s disease (Suzuki et al., 2005; Elfinger et al., 2007; Liu et al., 2013; Meng et al., 2018).

Poly(ethylene glycol)–poly(lactic-co-glycolic acid) (PEG-PLGA) copolymers have received FDA approval for their applications in drug delivery because of its biodegradability, biocompatibility, and versatility. PEG-PLGA nanoparticles were successfully prepared and it is found that PEG-PLGA nanoparticles accelerated blood clearance (Saadati et al., 2013). PLGA-NPs (carrier systems) were also containing excellent permeability and solubilizing effect (Ahmad, 2017). The potential of PEG-PLGA copolymers as targeting carriers in DDS is gradually being discovered and will be applied more widely.

In line with these observations, in our present study, we prepared borneol and Lf co-modified nanoparticles (Lf-BNPs) encapsulated dopamine, with enhanced permeability and striatum-specificity, and evaluated the improvement in PD treatment efficacy following intranasal administration. Cytotoxicity and cellular uptake of the formulations were evaluated by SH-SY5Y and 16HBE cells in vitro, and pharmacokinetic and pharmacodynamic studies of the prepared formulations were also performed.

## Materials and methods

### Materials and animals

Borneol was supplied from Yongye Biotechnology Co., Ltd. (Shanghai, China). Dopamine hydrochloride was a gift from Shanghai Keshun Biotechnology Co., Ltd. (Shanghai, China). 2-iminothiolane (Traut’s reagent), bovine Lf, 3-[4,5-dimethylthiazol-2-yl]-2,5-diphenyltetrazolium bromide (MTT), apomorphine, ascorbic acid, and 6-hydroxydopamine (6-OHDA) were purchased from Sigma-Aldrich (St. Louis, MO, USA). PEG-PLGA-maleimide (mal-PEG-PLGA; MW: 3.4–20 kDa) and methoxyPEG-PLGA (mPEG-PLGA; MW: 2–20 kDa) were purchased from Polyscitech (West Lafayette, IN, USA). All other chemicals and reagents were of analytical reagent grade.

Sprague-Dawley rats (male 200 ± 20 g) were provided by the Experimental Animal Center of Luye Pharmaceutical Group and housed in a room kept at 22 ± 1 °C under standard 12 h light/dark cycles. All animal studies were performed according to the Guide for the Care and Use of Laboratory Animals and approved by the Animal Ethics Committee of Yantai University.

### Preparation of dopamine Lf-BNPs

Dopamine-loaded BNPs were prepared using the double emulsion solvent evaporation method (Shin et al., 2014; Pahuja et al., 2015). In brief, dopamine hydrochloride (50 mg/mL) was added dropwise to 90 mg mPEG-PLGA, 10 mg mal-PEG-PLGA and 10 mg borneol dissolved in 5 mL of dichloromethane and sonicated using a probe sonicator to generate primary water/oil emulsion. Then, the emulsion was added dropwise to aqueous solution of polyvinyl alcohol, followed by sonication to produce a water/oil/water emulsion. And this emulsion was magnetically stirred till complete volatilization of the organic solvent.

According to a previously reported method (Huwyler et al., 1996), Lf was thiolated for 1 h with Traut’s reagent. The product was purified using a Sephadex G25 column. Then the purified thiolated Lf was added to the prepared BNPs and incubated for 9 h to obtain the Lf-BNPs. The solution was then eluted to remove the unconjugated thiolated Lf by a Sepharose CL-4B column.

### Characterization of formulations

#### Characterizing dopamine-loaded nanoparticles

The particle size, zeta potential, and polydispersity index were measured by dynamic light scattering method using a Zetasizer Nano-ZS (Deisa TM Nano C; Beckman Coulter, Brea, CA, USA). The shape and surface morphology of Lf-BNPs were studied by transmission electron microscopy (TEM) (JEM-1400, JEOL Ltd., Japan) following staining with a 2% (w/v) sodium phosphotungstate solution.

#### Determination of entrapment efficiency

Entrapment efficiency was determined by high-performance liquid chromatography (HPLC) with a C18 reverse-phase column. The mobile phase was consisted of 2% methanol (v/v) and 98% PBS (v/v, pH 5.8), and the effluent was monitored at 280 nm. Briefly, total drug content (free + entrapped) of formulations was studied by dissolving the formulation in acetic acid and free drug content was separated by centrifugation. The entrapment efficiency was calculated using the following equation:

$$\text{Entrapment}\text{Efficiency}\;(\%)=\frac{\text{Total}\text{drug}-\text{Free}\text{drug}}{\text{Total}\text{drug}}\;\times \;100$$

#### *In vitro* release profile

Dopamine release from the formulations was determined using the dialysis method (Pahuja et al., 2015). Samples investigated encompassed dopamine-loaded NPs, Lf-NPs, Lf-BNPs, and drug solution. Samples were transferred to dialysis bags that were immersed in 30 mL phosphate-buffered saline (PBS, pH 7.4) and then placed in an incubator shaker with a water bath at 37 °C. At predetermined time points, aliquots (1 mL) were taken and replaced with the same amount of fresh PBS. The samples were analyzed in triplicate using HPLC at 280 nm. The standard curve of dopamine was used to calculate the cumulative release of dopamine.

### Cellular studies of NPs

#### *In vitro* assessment of cytotoxicity

The cytotoxicity of free drugs and prepared formulations was evaluated by the MTT assay method. In brief, SH-SY5Y and 16HBE cells were seeded at a density of 10^4^ cells/well in a 96-well plate and cultured for 24 h at 37 °C with 5% CO_2_. Then, cells were exposed to different concentrations of free dopamine (5–200 μg/mL) or the different NP formulations containing equivalent dopamine content for 24 h. Then, MTT (5 mg/mL) was added to per well and cells were incubated for 4 h. Dimethyl sulfoxide (200 μL) was then added to dissolve the formazan crystals, and the absorbance was measured at 570 nm on a Cytation 5 imaging reader (BioTek Instruments Inc., VT, USA).

#### Cellular uptake of NPs

Qualitative analyses of cellular internalization of Nile red-loaded NPs, Lf-NPs and Lf-BNPs were performed by fluorescence microscopy (Eclipse E400; Nikon Corporation, Tokyo, Japan), while quantitative analyses of coumarin-6-loaded NPs were performed by flow cytometry (BD Accuri C6; BD Biosciences, San Jose, CA, USA).

For fluorescence microscopy, SH-SY5Y and 16HBE cells were seeded in a 24-well plate at a density of 5 × 10^4^ cells/well and cultured for 24 h at 37 °C in a 5% CO_2_ environment. After incubation for 24 h, the cells were incubated with Nile red-loaded NPs (1 μg/mL) at different times. Subsequently, the cells were washed 3 times with PBS and fixed with 4% paraformaldehyde for 10 min. Finally, the cells were visualized by fluorescence microscopy.

For flow cytometry, SH-SY5Y and 16HBE cells were seeded in a 6-well plate at a density of 5 × 10^5^ cells/well and treated as described earlier. After incubation with the same concentrations (6 ng/mL) of coumarin-6-loaded NPs, Lf-NPs or Lf-BNPs for 15 min, 1 h, and 2 h, the cells were washed 3 times with PBS. Then the cells were trypsinized and centrifuged at 1500 rpm for 5 min. After resuspension in PBS, the cells were analyzed by flow cytometry.

### Pharmacokinetic study

#### Experimental procedure

For the pharmacokinetic study, Sprague-Dawley rats were divided into three groups and intranasally administered dopamine-loaded NPs (group I), Lf-NPs (group II), and Lf-BNPs (group III). Blood samples were collected for analysis by retro-orbital puncture at 0.25, 0.5, 1, 2, 4, 8, and 12 h post administration, and plasma was obtained after centrifugation. Then, the rats were sacrificed humanely at predetermined time points, and the brains were harvested and homogenized.

#### Sample analysis

Dopamine concentrations in the plasma and brain were measured by HPLC – tandem mass spectrometry (HPLC-MS/MS; AB Sciex API 4500 triple-quadrupole mass spectrometer, USA; SHIMADZU LC-30 A high-performance liquid chromatography, Japan). The chromatographic separation was carried out on a C18 column under the following conditions: acetonitrile: 0.1% formic acid (65:35, v/v) at a flow rate of 0.8 mL/min; sample injection volume, 3 μL; column temperature, 35 °C. Mass spectrometer was operated in the positive ion mode by multiple reaction monitoring of the transition of *m/z* 154/137 for dopamine. The pharmacokinetic parameters were calculated by DAS v2.0 software (Mathematical Pharmacology Professional Committee of China, China).

### Pharmacodynamics study

#### Experimental procedure

Sprague-Dawley rats were examined one day before experiment to confirm that there was no rotation behavior. For the stereotaxic surgery, rats were deeply anesthetized by chloral hydrate (350 mg/kg) and then placed in a NEUROSTAR stereotaxic apparatus (Stereo Drive, Germany) to achieve a flat skull position. A small hole was drilled at the following coordinates: lateral (L) 1.65 mm and antero-posterior (AP) 0.4 mm, from the bregma point. 6-OHDA solution (4 mg/mL in 1% ascorbic acid) was injected unilaterally into the striatal at 0.5 μL/min. Three weeks after the surgery, contralateral rotations were recorded after injection of apomorphine hydrochloride (2 mg/kg). Rats exhibiting rotations of more than 75 rotations per 15 min are considered to have developed PD (Afshin-Majd et al., 2017).

#### Treatments and behavioral testing

Parkinson’s disease rats were divided into four groups with five animals per group. PBS was administered intranasally as negative control. Rats were administered intranasally dopamine-loaded NPs, Lf-NPs, and Lf-BNPs, respectively. Rotations were recorded for 15 min on the 1st, 10th, and 20th days after apomorphine administration.

#### Determination of monoamine neurotransmitter level in the striatum

The rats were euthanized following the behavior tests. Striatum of the lesioned side was removed and homogenized. HPLC-MS/MS was used to measured dopamine and its metabolites (dihydroxyphenylacetic acid (DOPAC) and homovanillic acid (HVA)). Data are expressed as ng/g tissue weight.

#### Data analysis

All data are expressed as mean ± standard derivation (SD). Statistical analysis was performed by one-way ANOVA using SPSS 22.0 software (IBM Corp., Armonk, NY, USA). Statistically significant and highly significant differences are defined as *p* < .05 and *p* < .01, respectively.

## Results and discussion

### Preparation and characterization of nanoparticles

The particle size, zeta potential, polydispersity index, and entrapment efficiency of dopamine-loaded NPs, Lf-NPs, and Lf-BNPs were shown in Table 1. The morphology and size distribution of Lf-BNPs were determined by TEM, and the result was presented in Supporting Information (Figure S1). The TEM images showed that the NPs were spherical and had a smooth surface. It may have led to a smaller diameter compared with DLS, because the NPs were dehydrated and shrank during TEM preparation. The mean diameters of formulations were not more than 200 nm, which indicated that the prepared formulations are suitable for intranasal administration (Mistry et al., 2009). The mean diameter of NPs was about 100 nm but increased when modified with Lf. And also, the diameters of borneol-modified NPs differed. The zeta potential values were about −15 mV and slightly affected by the modification of borneol, demonstrating that the formulations were stable. A low value (<0.2) of polydispersity index revealed the uniformity in the size of particles. The entrapment efficiency was found to be dependent on the amount of polymer ratio (Ahmad, 2017). In this work, the entrapment efficiency of formulations was more than 25%, and there was no significant difference between the prepared nanoparticles.

**Table 1:** Characterization of dopamine-loaded nanoparticles.

Formulation	Particle size, nm	Zeta potential, mV	Polydispersity index	Entrapment efficiency, %
NPs	101.9 ± 4.8	−16.3 ± 0.23	0.061 ± 0.029	30.99 ± 3.49
Lf-NPs	158.4 ± 5.4	−19.9 ± 1.01	0.113 ± 0.034	28.64 ± 3.68
Lf-BNPs	175.3 ± 9.6	−15.7 ± 0.86	0.129 ± 0.011	25.43 ± 5.32

### *In vitro* release profile

The release kinetics was studied for 5 days in PBS (pH 7.4) at 37 °C. The cumulative release percentage of dopamine from dopamine solution, dopamine-loaded NPs, Lf-NPs, and Lf-BNPs at many time points was shown in Supporting Information (Figure S2). Free dopamine was rapidly released within 4 h (91.24 ± 4.03%). In contrast, the release profiles all exhibited the sustained release characteristics of dopamine from NPs. The cumulative release percentage of dopamine from these formulations can be up 70% in 72 h. NPs and Lf-NPs showed higher release rates than Lf-BNPs, and there were no significant differences when compared with the borneol modification of NPs.

### *In vitro* assessment of cytotoxicity

**Figure 1.**
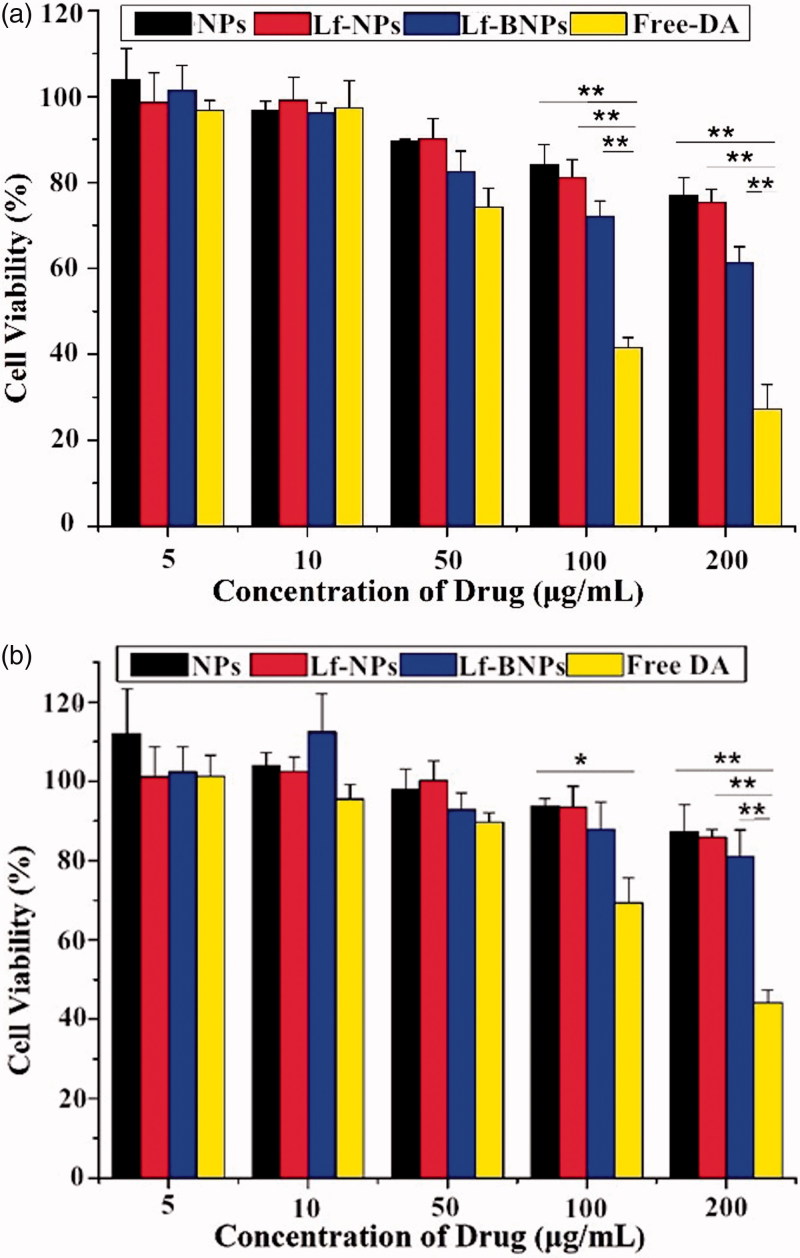
In vitro cytotoxicity of free dopamine and corresponding concentrations of dopamine-loaded NPs, Lf-NPs, and Lf-BNPs on 16HBE (a) and SH-SY5Y cells (b). Cells were exposed to different concentrations of free dopamine, NPs, Lf-NPs, and Lf-BNPs for 24 h and cell viability was measured. Values represent the mean ± SD (*n* = 3). *p < .05; **p < .01 (versus Free dopamine group).

*In vitro* cytotoxicity assessment of prepared formulations is essential for evaluating the safety after intranasal administration. As shown in Figure 1, exposure to free dopamine at 100 and 200 µg/ml for 24 h induced significant cell death both in SH-SY5Y and 16HBE cells. In contrast, dopamine-loaded NPs, Lf-NPs, and Lf-BNPs did not cause significant cell death at any of the tested concentrations. Moreover, SH-SY5Y and 16HBE cells exposed to dopamine-free NPs, Lf-NPs, and Lf-BNPs did not show a significant decrease in viability at any of the tested concentrations (data not shown). These results indicated that polymer-encapsulated dopamine formulations showed reduced toxicity compared with free dopamine. There was no significant difference in the NP, Lf-NP, and Lf-BNP groups, indicating that both borneol and Lf were nontoxic.

### Cellular uptake of NPs

**Figure 2.**
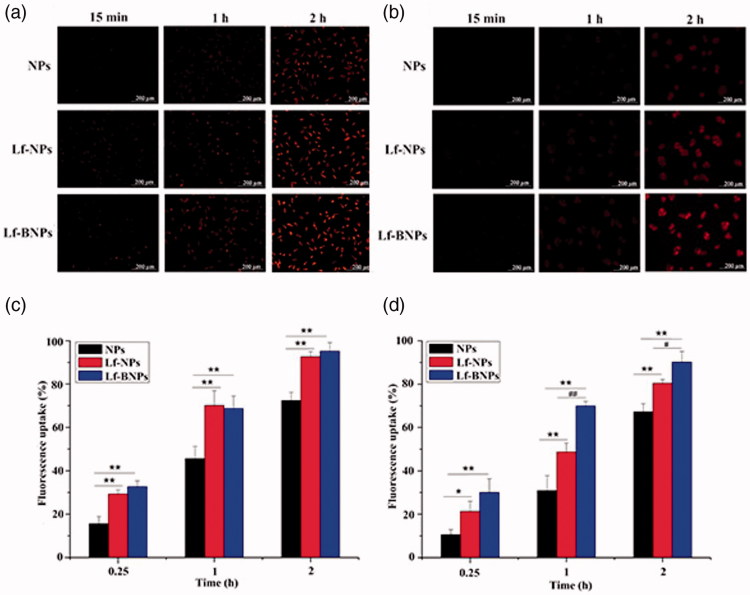
Cellular uptake of NPs. Fluorescence microscopy images of cellular uptake of Nile red NPs in SH-SY5Y (a) and 16HBE cells (b). Fluorescence intensity on flow cytometry of coumarin-6 NPs in SH-SY5Y (c) and 16HBE cells (d). Values are expressed as mean ± SD (*n* = 3). *p < .05; **p < .01 (versus NPs group); ^#^*p* < .05 (versus Lf-NPs group).

Cellular uptake of NPs in SH-SY5Y and 16HBE cells was qualitatively evaluated using fluorescence microscopy (Figure 2(a,b)) and quantitatively analyzed by flow cytometry (Figure 2(c,d)). For SH-SY5Y cells and 16HBE cells, the fluorescence intensity of Lf-NPs was higher than that of NPs. The same results were obtained from quantitative analysis, in which the mean fluorescence intensity of Lf-NPs was significantly higher than that of NPs (*p* < .05). The Lf receptor is overexpressed on the apical surface of respiratory epithelial cells and in the capillaries and neurons related to neurodegenerative diseases, thus Lf modification of NPs increasing cellular uptake of SH-SY5Y cells and 16HBE cells. There was no obviously difference between Lf-BNPs and Lf-NPs for SH-SY5Y cells, while the mean fluorescence of Lf-BNPs was significantly higher than that of Lf-NPs (*p* < .05) for 16HBE cells. These results demonstrated that borneol modification can promote the cellular uptake of 16HBE.

### Pharmacokinetic study

Plasma and brain dopamine levels post intranasal administration of dopamine-loaded NPs, Lf-NPs, and Lf-BNPs at various pre-determined intervals up to 12 h in rats are shown in Figure 4. And various pharmacokinetic parameters were calculated and are represented in Table 2. The concentrations reported were the measured concentrations minus the concentrations of endogenous dopamine in blood and brain.

**Table 2:** Pharmacokinetic parameters of dopamine-loaded nanoparticles after intranasal administration in rats in the brain and blood.

Formulation	Tissue	*C*_max_ (ng/mL or ng/g)	*T*_max_ (h)	Ke (h^−1^)	AUC_0–12 h_ (ng.h/mL or ng.h/g)	AUC_0–∞_ (ng.h/mL or ng.h/g)
NPs	Blood	160.2 ± 16.5	1	0.29 ± 0.08	686.0 ± 73.4	714.5 ± 61.3
	Brain	149.3 ± 21.7	1	0.15 ± 0.04	830.6 ± 108.9	1070.0 ± 188.3
Lf-NPs	Blood	168.3 ± 17.5	1	0.29 ± 0.04	758.3 ± 116.9	784.1 ± 128.8
	Brain	200.4 ± 13.7	1	0.18 ± 0.07	1341.8 ± 109.8*	1604.0 ± 112.3
Lf-BNPs	Blood	175.3 ± 16.8	0.5	0.37 ± 0.02	668.1 ± 102.4	675.3 ± 105.2
	Brain	219.4 ± 13.3	0.25	0.17 ± 0.08	1696.4 ± 119.3**^#^	1943.8 ± 202.5

Values are mean ± SD (*n* = 3). *p < .05; **p < .01 (versus NP group); ^#^*p* < .05 (versus Lf-NP group).

**Figure 3.**
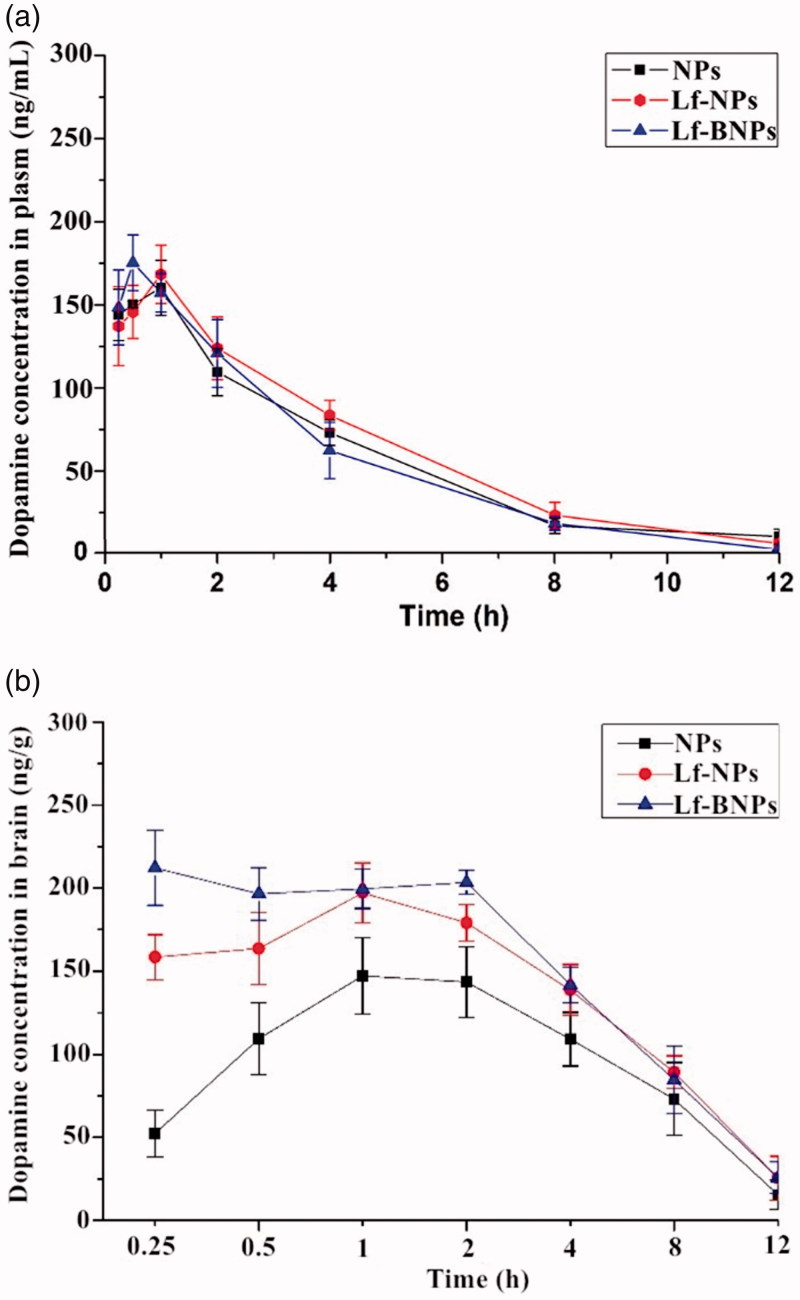
(a) Blood and (b) brain concentration-time profiles of dopamine following intranasal administration of dopamine-loaded NPs, Lf-NPs, and Lf-BNPs. Data represent the mean ± SD.

The plasma concentration-time curves of NP, Lf-NP, and Lf-BNP groups were similar (Figure 3(a)), indicating that borneol and Lf co-modification did not impair the circulation characteristics of PEG. The dopamine concentrations in the brain following nose-to-brain delivery of dopamine-loaded Lf-NPs and Lf-BNPs were significantly higher at all time points than that following the administration of dopamine NPs (maximum concentrations were 200.4 ± 13.7 at 1 h and 219.4 ± 13.3 at 0.25 h, respectively). The dopamine concentrations of Lf-BNPs were found to be significantly higher than that of Lf-NPs at 0.25 h and 0.5 h, suggesting that borneol-modified NPs could be effective in promoting the delivery drugs into the brain. In the first 0.25 h post intranasal administration, the brain/blood ratios of 0.36 ± 0.10, 1.18 ± 0.29, and 1.44 ± 0.15 of NPs, Lf-NPs, and Lf-BNPs, respectively, were indicative of intranasal delivery circumventing the blood-brain barrier, thus demonstrating the superiority of direct nose-to-brain transport of dopamine by Lf-BNPs.

The peak plasma concentrations of dopamine in blood post intranasal administration of dopamine-loaded NPs, Lf-NPs, and Lf-BNPs were 160.2 ± 16.5, 168.3 ± 17.5, and 175.3 ± 16.8 ng/mL with *t*_max_ of 1, 1, and 0.5 h, respectively, and the peak plasma concentrations of dopamine in the brain post intranasal administration of dopamine-loaded NPs, Lf-NPs, and Lf-BNPs were 149.3 ± 21.7, 200.4 ± 13.7, and 219.4 ± 13.3 ng/g with *t*_max_ of 1, 1, and 0.25 h, respectively. The lower *t*_max_ values for the brain (0.25 h) than that for blood (0.5 h) may also be attributed to the preferential nose-to-brain delivery post-nose-to-brain delivery of dopamine Lf-BNPs. The AUC_0–12 h_ (1696.4 ± 119.3 ng.h/g) of Lf-BNPs was higher than that of NPs and Lf-NPs. The intranasal delivery of dopamine Lf-NPs promoted dopamine concentration in the brain compared with the equivalent dose of NPs, and intranasal delivery of Lf-BNPs enhanced the delivery of dopamine to the brain compared with NPs and Lf-NPs.

The results demonstrated that Lf and borneol co-modified NPs significantly increased dopamine delivery to the brain via the intranasal route. In the previous report, drug uptake from nose to the brain mainly occurs via three different pathways: a systemic pathway of absorption and subsequently into the brain by crossing the blood-brain barrier, and a direct pathway from nasal mucosa epithelium into the brain mainly along the trigeminal or olfactory nerves that bypass the blood-brain barrier (Wen et al., 2011; Lalani et al., 2014). Therefore, borneol and Lf co-modified NPs not only enhance the penetration and transport of dopamine through nasal mucosa, but also enhance the drug delivery by opening the tight junctions of the blood–brain barrier.

### Pharmacodynamics study

In this experiment, dopamine-loaded NPs, Lf-NPs, and Lf-BNPs were administered intranasally for evaluating therapeutic efficacy in 6-OHDA-induced PD rats. The contralateral rotation behavior was used to assess the therapeutic efficacy of prepared formulations. Apomorphine-induced rotation is considered as a quantitative measure of the severity of striatal lesion (Ungerstedt, 1971). Evaluation of contralateral rotations before surgery exhibited no statistically significant differences among the experimental groups. However, 6-OHDA-lesioned rats showed significant contralateral rotations compared with normal rats (data not shown).

**Figure 4.**
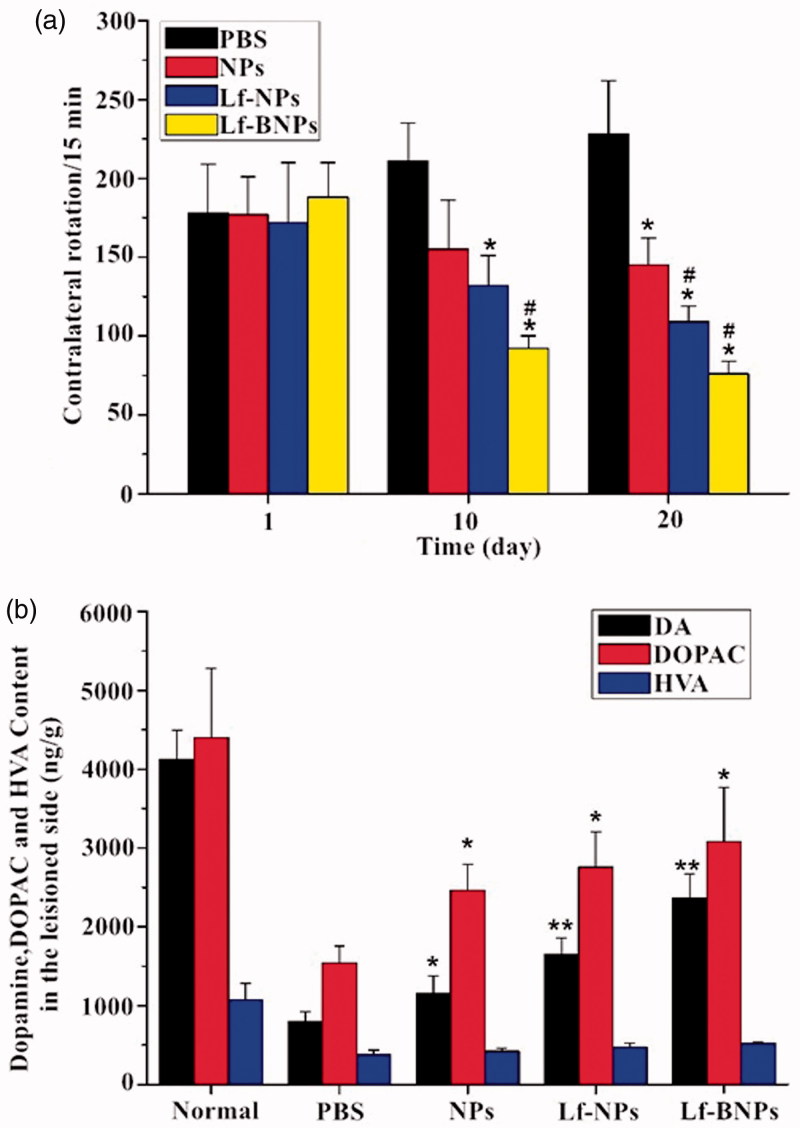
(a) The number of apomorphine-induced contralateral rotations per 15 min in 6-hydroxydopamine-lesioned rats treated with PBS, dopamine-loaded NPs, Lf-NPs, and Lf-BNPs, respectively. (b) Monoamine neurotransmitter levels in the striatum of the lesioned side treated with PBS, dopamine-loaded NPs, Lf-NPs, and Lf-BNPs after 20 days. Values are expressed as mean ± SD. *n* = 5 for each group. *p < .05; **p < .01 (versus PBS group); ^#^*p* < .05 (versus NP group).

The results of the contralateral rotation behavior test are shown in Figure 4(a). After 20 days of administration, the number of contralateral rotations in the PBS group gradually increased, which demonstrated the deterioration of striatum. In contrast, the rotational behavior in nanoformulation groups exhibited a consistent decline to different degrees during the treatment. The apomorphine-induced rotations in the Lf-NP and Lf-BNP groups were much lower than that of the PBS group on days 10 and 20, while the rotations in the NP group were not significantly different (*p* > .05) compared with those in the PBS group on day 10. Moreover, the apomorphine-induced rotations in the Lf-BNP group were statistically significant difference from the Lf-NP group on day 20 (*p* < .05).

In order to evaluate the change in the content of dopamine in the striatum, an HPLC-MS/MS method was used to detect dopamine and its metabolites in the striatum. After day 20, the levels of dopamine, DOPAC and HVA in the PBS group were significantly lower than that in the normal group, while post-treatment with dopamine Lf-BNPs, the contents of dopamine, DOPAC, and HVA were significantly higher in comparison with the PBS group (Figure 3(b)). These results indicated that dopamine Lf-BNPs effectively restored dopamine level in the lesioned striatum of 6-OHDA-induced PD rats. These results are in accordance with behavioral test results.

Our finding revealed that Lf-NPs and Lf-BNPs can deliver effective doses of dopamine into the lesioned striatum after intranasal administration. Moreover, our results indicated that the therapeutic efficacy of dopamine Lf-NPs was better than that of dopamine NPs, and the therapeutic efficacy of dopamine Lf-BNPs was greater than that of dopamine Lf-NPs, indicating that modification with borneol and Lf can deliver more drugs into the striatum to achieve ideal therapeutic efficacy.

## Conclusion

In the present study, we proposed borneol and Lf co-modified nanoparticles encapsulated drug to be an effective nose-to-brain drug delivery system for enhancing dopamine delivery into the brain for the treatment of PD. Cell viability evaluation results showed the relatively low toxicity of NP formulations. Qualitative and quantitative cellular uptake experiments demonstrated that Lf modification of NPs increased cellular uptake of SH-SY5Y cells and 16HBE cells, and borneol modification can promote the cellular uptake of 16HBE. Pharmacokinetic studies showed significantly increased absorption of dopamine Lf-BNPs into the brain after nose-to-brain delivery than that of dopamine NPs, which demonstrated that borneol and Lf co-modification could together enhance drug transport to the brain via intranasal administration. Pharmacodynamic study indicated that 6-OHDA-induced PD rats treated with dopamine Lf-BNPs showed decreased apomorphine-induced contralateral rotations and increased dopamine content in the lesioned striatum. In conclusion, the results demonstrated that the drug delivery system in this study could provide an effective noninvasive approach to encourage the access of dopamine to the brain for treatment of PD.

## Supplementary Material

Supplemental Material
