# Supplementary material for: Brain-targeted intranasal delivery of dopamine with borneol and lactoferrin co-modified nanoparticles for treating Parkinson’s disease
Source: Drug Deliv. 2019 Jul 10;26(1):700–7. doi: 10.1080/10717544.2019.1636420 (PMC7577045; doi:10.1080/10717544.2019.1636420)
Supplement: Supplemental Material [file IDRD_A_1636420_SM4496.docx]

Supporting Information

for

**Brain-targeted intranasal delivery of dopamine with borneol and lactoferrin co-modified nanoparticles for treating Parkinson’s disease**

Shengnan Tang^a*^, Aiping Wang^a*^, Xiuju Yan^a^, Liuxiang Chu^a^, Xiucheng Yang^a^, Yina Song^a^, Kaoxiang Sun^a^, Xin Yu^a^, Rongxia Liu^a^, Zimei Wu^a^, Peng Xue^b^

^a^ School of Pharmacy, Collaborative Innovation Center of Advanced Drug Delivery System and Biotech Drugs in Universities of Shandong, Key Laboratory of Molecular Pharmacology and Drug Evaluation (Yantai University), Ministry of Education, Yantai University, Yantai, Shandong Province, People’s Republic of China

^b^ State Key Laboratory of Long-Acting and Targeting Drug Delivery System, Shandong Luye Pharmaceutical Co., Ltd, Yantai, Shandong Province, People’s Republic of China

*These authors contributed equally to this work.

Corresponding author:

Aiping Wang

School of Pharmacy, Yantai University, No 30 Qingquan road, Yantai, 264005, Shandong Province, People’s Republic of China

Tel: 86 535 3946458, Email: wangaiping@luye.com


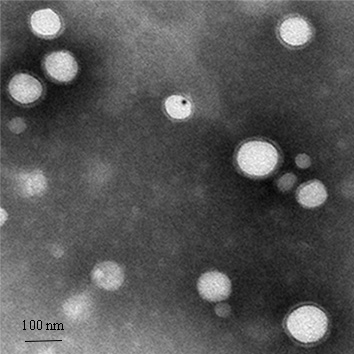


**Figure S1**. Transmission Electron Microscopy (TEM) images of Lf-BNPs (Scare bar 100 nm).


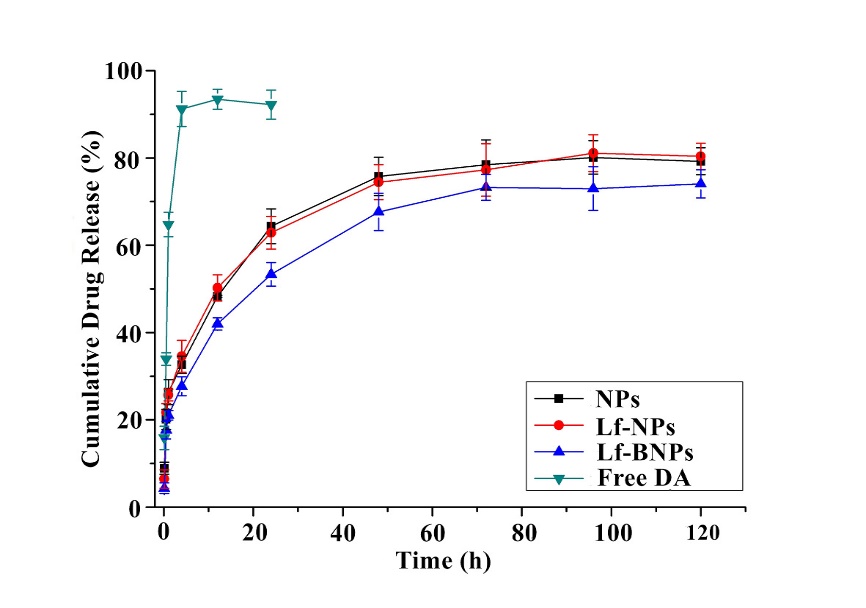


**Figure S2**. Release profile of dopamine in PBS from free dopamine, dopamine-loaded NPs, Lf-NPs, and Lf-BNPs (n=3, mean ± SD).
